# Hepatic Aryl hydrocarbon Receptor Nuclear Translocator (ARNT) regulates metabolism in mice

**DOI:** 10.1371/journal.pone.0186543

**Published:** 2017-11-30

**Authors:** Christopher H. Scott, Kuan-Minn Cha, Jason Ngai, Changtao Jiang, Kim Cheng, Rebecca A. Stokes, Kenneth W. K. Ho, Jacob George, Frank J. Gonzalez, Jenny E. Gunton

**Affiliations:** 1 The Westmead Institute for Medical Research, The University of Sydney, Sydney, NSW. Australia; 2 Diabetes and Transcription Factors Group, Garvan Institute of Medical Research, Sydney, NSW, Australia; 3 Laboratory of Metabolism, National Cancer Institute, Bethesda, Maryland, United States of America; 4 Storr Liver Unit, Westmead Institute for Medical Research, The University of Sydney, Sydney, NSW. Australia; 5 St. Vincent’s Clinical School, University of NSW, Sydney, NSW, Australia; 6 Department of Diabetes and Endocrinology, Westmead Hospital, Sydney, NSW Australia; INRA, FRANCE

## Abstract

**Background & aims:**

Aryl hydrocarbon Receptor Nuclear Translocator (ARNT) and its partners hypoxia-inducible factors (HIF)-1α and HIF-2α are candidate factors for the well-known link between the liver, metabolic dysfunction and elevation in circulating lipids and glucose. *Methods*: Hepatocyte-specific *ARNT*-null (LARNT), *HIF-1α*-null (LHIF1α) and *HIF-2α*-null (LHIF2α) mice were created.

**Results:**

LARNT mice had increased fasting glucose, impaired glucose tolerance, increased glucose production, raised post-prandial serum triglycerides (TG) and markedly lower hepatic ATP versus littermate controls. There was increased expression of *G6Pase*, *Chrebp*, *Fas* and *Scd-1* mRNAs in LARNT animals. Surprisingly, LHIF1α and LHIF2α mice exhibited no alterations in any metabolic parameter assessed.

**Conclusions:**

These results provide convincing evidence that reduced hepatic ARNT can contribute to inappropriate hepatic glucose production and post-prandial dyslipidaemia. Hepatic ARNT may be a novel therapeutic target for improving post-prandial hypertriglyceridemia and glucose homeostasis.

## Introduction

The liver is central in the homeostatic regulation of glucose and lipids. Hepatic dysfunction plays a key role in the pathogenesis of metabolic syndrome, dyslipidaemia and type 2 diabetes. In concordance with this a number of animal models have demonstrated that liver dysfunction induces components of the metabolic syndrome and T2D [1–3]. Non alcoholic fatty liver disease (NAFLD) is the accumulation of macroscopically visible lipid in hepatocytes in the absence of excess alcohol consumption (<20g of ethanol/day) [4]. Histologically NALFD is defined by the accumulation of fat in the liver >5% of liver weight [5]. The spectrum of NAFLD includes a range of pathological features from mild steatosis to non-alcoholic steatohepatitis (NASH) and cirrhosis with increasing levels of liver dysfunction [6]. NAFLD is cited as the liver component of the metabolic syndrome (MetS), with a similar prevalence of 34%, and is predicted to become the leading of chronic liver disease and reason for liver transplants [7]. Most NAFLD patients have increased liver fat content alone but some (10%-30%) go on to develop inflammation and fibrosis or non alcoholic steato hepatitis (NASH) [8, 9]. NASH can in turn lead to increasing liver fibrosis and cirrhosis, liver failure and hepatic carcinoma (HCC) [10, 11]. Like the metabolic syndrome weight loss has been shown to be effective in reducing markers of disease [12]. However the efficacy of such interventions is limited by patient compliance and relapse of disease is common [13]. Thiazolidedione, Metformin, and HMG Co-A reductase inhibitors (statins) have been used with some success, although further studies are needed to assess long term safety and clinical outcomes [12, 14, 15].

One attractive candidate which may be involved in metabolic liver disease is the multifunctional transcription factor, Aryl hydrocarbon Receptor Nuclear Translocator (ARNT). It heterodimerises with other bHLH/PAS family members including Hypoxia-Inducible Factor-1-α (HIF-1α), Hypoxia-Inducible Factor-2-α (HIF-2α) and Aryl hydrocarbon Receptor (AhR) to form active transcription complexes which regulate genes involved in hypoxic-responses, cell survival, proliferation, glycolysis, angiogenesis and xenobiotic responses [16–19]. The endogenous ligands for AhR remain highly contentious; bile acids, cAMP and tryptophan metabolites are reported to be potential candidates, while a recent paper points to other ligands in breakdown products from cruciferous vegetables [20–23].

Interestingly, acute increases in hepatic HIF-1α and HIF-2α induced by adenoviral-cre mediated deletion of *von Hipplel-Lindau* (*Vhl*) factor cause fatty liver and hypoglycemia [24]. Either deletion or activation of AhR causes hepatosteatosis [25–27], and deletion of hepatic HIF-1α impairs glucose tolerance in mice given a high fat/sucrose diet [28].

Expression of *ARNT* in liver and pancreatic islets is decreased in patients with T2D [16, 29]. We have previously shown that short-term adenovirus-induced hepatic *Arnt* deletion in mice increased hepatic glucose production (HGP) and impaired glucose tolerance [29]. What remained unclear were the consequences of long-term decrease in hepatic ARNT, and which partner was important for the effects.

Hepatocyte specific *ARNT*-null (LARNT), hepatocyte specific *HIF-1α*–null (LHIF1α) and hepatocyte specific *HIF-2α*–null (LHIF2α) mice were created. The phenotype of LARNT mice included markedly decreased hepatic ATP, increased fasting glucose, increased HGP, defective glucose tolerance and higher post-prandial serum TG. The phenotype of LHIF1α and LHIF2α mice were unremarkable.

## Research design and methods

### Animal studies

Floxed ARNT, HIF1-α and HIF2-α mice were created as previously described [16, 30, 31]. Hepatocyte specific mice were created by breeding these mice with Albumin-Cre mice (kindly provided by David James, Garvan Institute) to produce LARNT, LHIF1 LHIF2 and their respective floxed-control littermate offspring. All mice were on an inbred C57Bl/6 background and housed in a facility with 12 hour light /dark cycle, an ambient temperature of ~22°C and ad libatum access to food. All procedures were approved by the Garvan Animal Ethics Committee or the Westmead Animal Ethics Committee. Mice were sacrificed after being anaesthetized with ketamine+xylazine.

### Physiological testing

Blood samples and physiological test samples were collected after an overnight fast (16 hours). For glucose tolerance tests (GTT) and insulin tolerance tests (ITT), mice were fasted then glucose (2g/kg body weight, Sigma-Aldrich, St. Louis, USA) or insulin (0.25U/kg Actrapid, Novo Nordisk, Sydney Australia) was given by intraperitoneal injection (IP). A tail nick was made and glucose levels were measured at the time-points shown using an Optium glucometer (Abbot Diabetes Care, Doncaster, Australia). For the pyruvate challenge test (PCT), mice were fasted and 2g/kg of pyruvate (Sigma-Aldrich, St. Louis, USA) dissolved in phosphate-buffered saline was given IP. Mice were sacrificed after an overnight fast unless otherwise specified, at least 1 week after the last physiological test. Livers were divided for formalin fixation or snap-freezing in liquid nitrogen for gene expression and lipid studies with the same lobe for each use, every time.

### Gene expression analysis

Liver was homogenized in RLT buffer (Qiagen, Valencia, USA). RNA was isolated and cDNA was synthesized as previously described [32, 33]. Real-time PCR was performed using specific primers and Sybr Green PCR master mix (Applied Biosystems, Melbourne Australia), and amplification was performed in an ABI7900 light-cycler (Applied Biosystems, Melbourne Australia). Results were corrected for expression of the housekeeping gene TATA-box binding-protein (TBP). Primers are shown in Table 1.

**Table 1:** Primer sequences.

*Gene name*	Forward	Reverse
*F16bp*	gaccctgccatcaatgagta	gttggcggggtataaaaaga
*G6pase*	tcggagactggttcaacctc	acaggtgacagggaactgct
*Fas*	gaggacactcaagtggctga	gtgaggttgctgtcgtctgt
*Scd-1*	cctgcggatcttccttatca	gtcggcgtgtgtttctgag
*Hmgs*	gccgtgaactgggtcgaa	gcatatatagcaatgtctcctgcaa
*Hmgr*	caaagtttgccctcagttca	gtgccaactccaatcacaag
*Pgc1a*	gtcaacagcaaaagccacaa	tctggggtcagaggaagaga
*Ppara*	tggcgtacgacaagtgtgat	gtttgcaaagcctgggatag
*Pparg*	gaataccaaagtgcgatcaaagta	ccaaacctgatggcattgtgagac
*Srebp1-c*	gagccatggattgcacattt	ctcaggagagttggcacctg
*Cpt-1*	cttccatgactcggctcttc	agcttgaacctctgctctgc
*Insr*	taccgcattgagctgcaggc	aagacaaagatgaggggtcc
*ARNT*	tctccctcccagatgatgac	caatgttgtgtcgggagatg
*Glut 1*	acctatggccaaggacacac	ctggtctcaggcaaggaaag
*Glut 2*	catgctgagctctgctgaag	acagtccaacggatccactc
*GK*	gagatggatgtggcaat	accagctccacacttctgat
*Chrebp*	gcatcctcatccgaccttta	caagaacagcaacgagtaccg
*Pepck*	ctaacttggccatgatgaacc	cttcactgaggtgccaggag
*Irs2*	gtagttcaggtcgcctctgc	ttgggaccaccactcctaag
*Akt2*	tttgtgttcccttccctgtc	tcactctccatcctcccaac

### Liver histology

Liver was dissected from floxed-control and LARNT mice and the left lobe from each mouse was fixed in 10% buffered formalin. Tissue was paraffin-embedded and 5μm sections were stained with hematoxylin and eosin (H&E) or Sirius Red according to standard protocols.

### Insulin and triglyceride assays

Insulin was measured using the Crystal Chem (Chicago, USA) ELISA kit as per the manufacturer’s instructions. Serum and liver triglyceride content were assayed using the Roche triglyceride kit (GPO-PAP, Mannheim, Germany). Liver was homogenized (30-40mg per mouse) and used to determine total triglyceride content which was expressed as μg/mg of liver. Lipid oxidation.

Primary hepatocytes were isolated as previously described [34] and cultured in 25cm^2^ flasks. They were washed and incubated in Kreb’s buffer plus 0.25% fatty acid free BSA (Sigma-Aldrich, St. Louis, USA) with 6mM glucose, 0.125mM palmitate and 0.25μCi/ml of [1-^14^C]-palmitic acid (GE Healthcare, Port Washington, USA). Filter paper soaked in 5% KOH was suspended over the cells and the flasks sealed shut. Cells were incubated at 37°C with 5% CO_2_ for 24 hours and the reaction stopped with 500μl of 40% perchloric acid. Filter paper radioactivity was counted in 5ml Microscint-20 (Perkin Elmer, Waltham, USA) using an LS 6500 Scintillation Counter (Beckman Coulter, Brea, USA) and results were corrected for total protein.

### ATP assay

ATP content of liver was measured as previously reported [33] and is expressed corrected for total protein.

### Statistical analysis:

Data was analysed using a 2-tailed Student’s t-test unless otherwise stated. P-values for statistics were calculated in Excel (students t-test) or Prism (ANOVA) and a p-value of <0.05 was considered significant. Data is mean ± SEM unless otherwise stated.

## Results

### Fasting glucose levels were increased in LARNT mice

**Fig 1:**
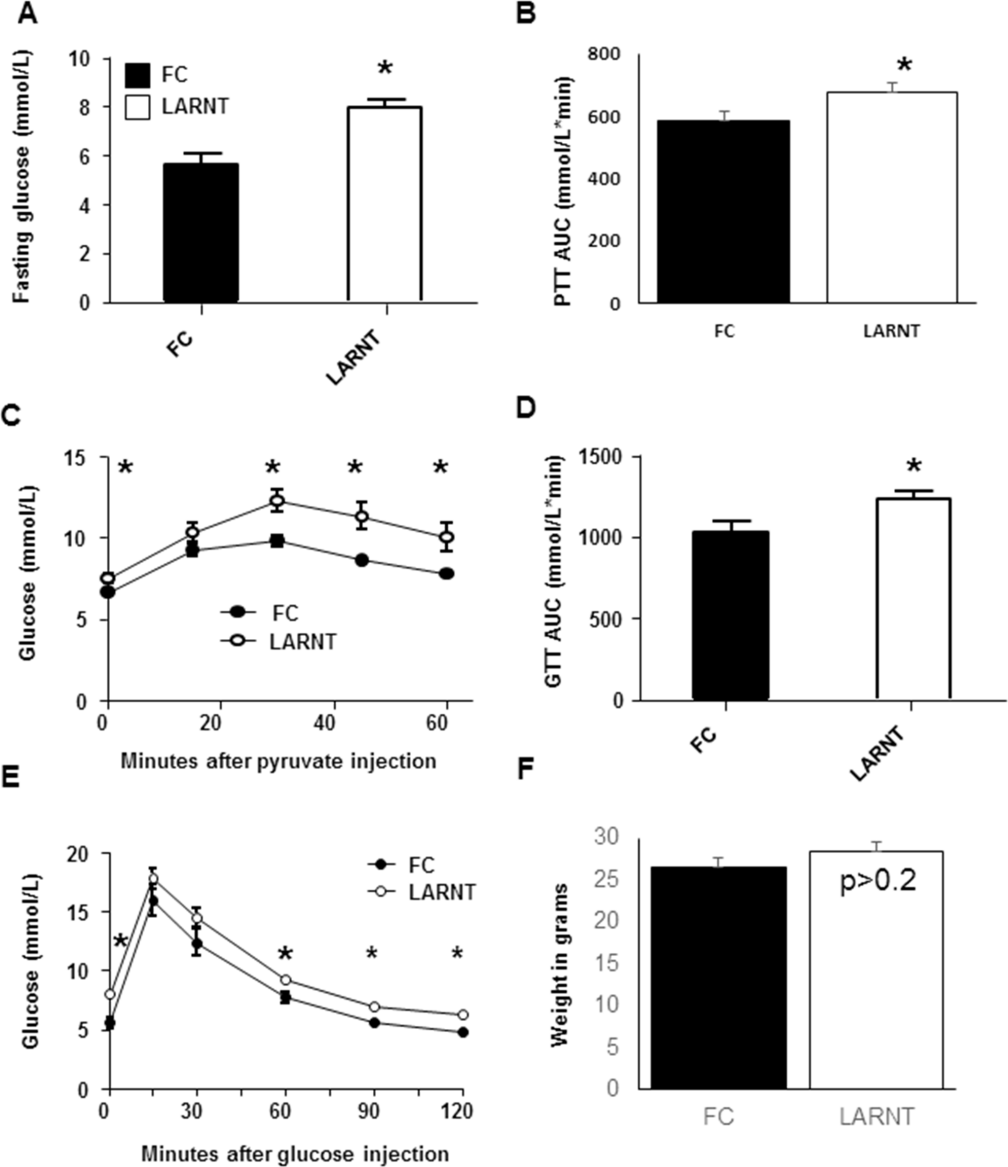
Hepatocyte specific ARNT knockout chow fed (LARNT) mice have impaired glucose metabolism. Results for floxed control (FC) mice are shown in black and LARNT mice in white (bars or circles) **(A)** Fasting blood glucose was higher in LARNT mice at 12 weeks. **(B)** Area under the curve (AUC) of pyruvate challenge tests (PCT) in FC and LARNT mice at 18weeks. **(C)** PCTs. **(D)** Glucose tolerance tests (GTT) AUC in FC and LARNT at 12 weeks. **(E)** GTT curves in FC and LARNT. **(F)** Body weight of FC and LARNT mice at 18–21 weeks. Mean±SEM, * = p<0.05. N = 5–13 per group.

People with diabetes have increased fasting glucose levels. Hepatic dysfunction and inappropriate hepatic glucose production (HGP) is a significant contributor to this. Fasting glucose was measured in LARNT animals. When compared with floxed controls (FC), fasting glucose levels were 42% higher in LARNT mice (p<0.001, **Fig 1A**).

### Serum glucose levels were increased in LARNT mice following pyruvate challenge testing (PCT)

To examine the effect of selective hepatocyte *ARNT* deletion on HGP, PCTs were performed. Fasting glucose levels were again higher in LARNT mice, as were the levels after pyruvate loading (AUC for the increase above baseline shown in **Fig 1B**, p = 0.021 and curves shown in **Fig 1C** p<0.005 by ANOVA for repeated measures).

### Glucose tolerance was impaired in LARNT mice

A key feature of T2D and metabolic syndrome is abnormal glucose tolerance. We found that hepatocyte-specific deletion of ARNT caused mildly but significantly elevated glucose during glucose tolerance testing compared to their littermate controls AUC was ~20% higher, and is shown in **Fig 1D**, p0.026. Glucose tolerance curves are shown in **Fig 1E.**

Variable changes in whole-body insulin sensitivity occurred in LARNT mice.

**Fig 2:**
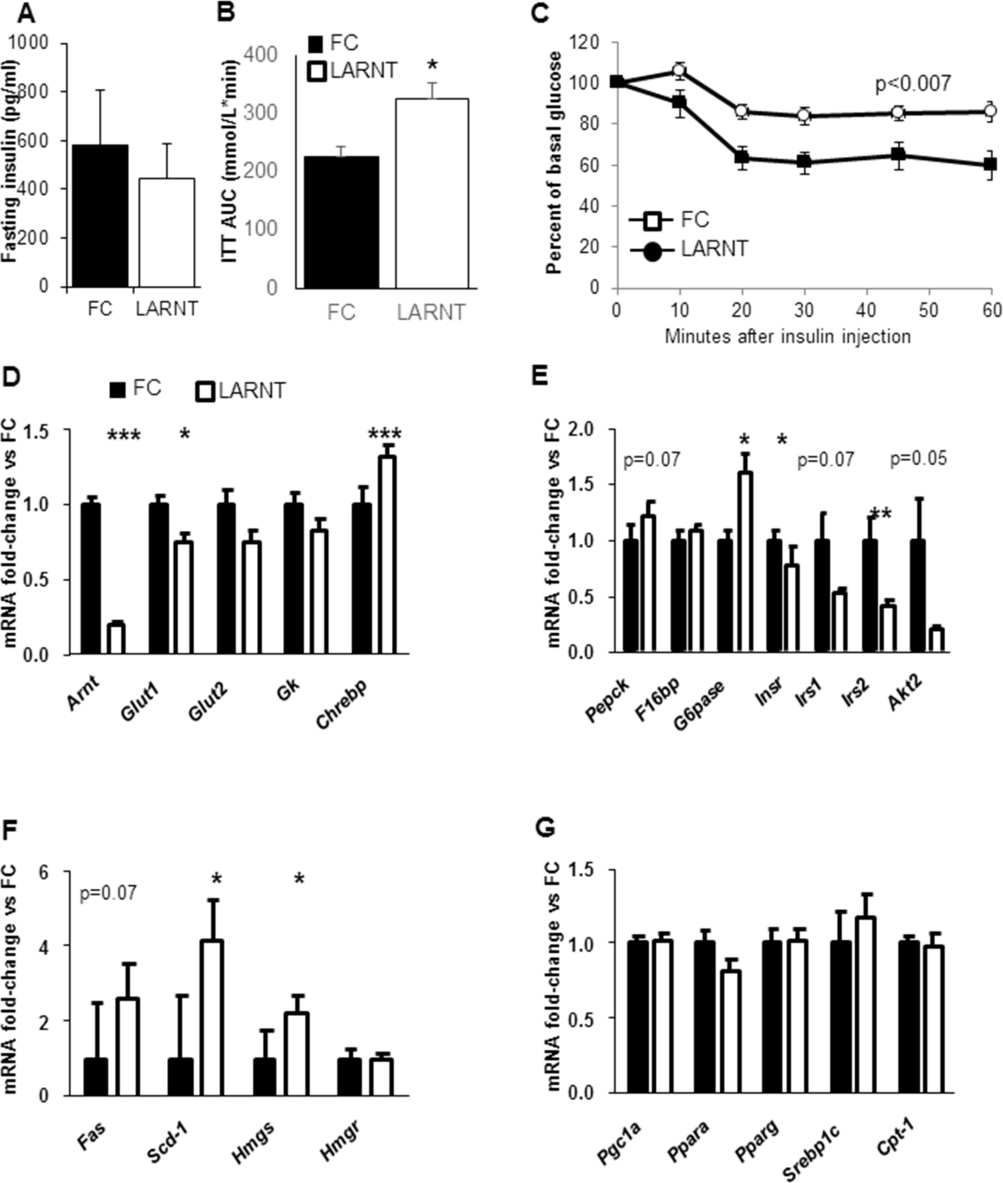
Hepatocyte ARNT deletion alters hepatic glucose production and gene expression. Results for floxed controls (FC) are shown in black and LARNT mice in white (bars or circles). **(A)** Fasting serum insulin levels in FC and LARNT mice at 20 weeks (median and 95% CI). **(B)** Insulin tolerance tests (ITT) Area Under the Curve (AUC) at 13 weeks. **(C)** ITT curves; levels are expressed as % of baseline glucose level at 0 minutes. **(D-G)** Fold changes in LARNT versus FC mice. * = p<0.05 ** = p<0.01, *** = p<0.001, N = 5–6.

There were no differences in body weight at 18 weeks of age (**Fig 1F**), and fasting serum insulin did not differ between LARNT and FC mice (Median and 95% CI, **Fig 2A**). To assess whole-body insulin sensitivity, insulin tolerance tests (ITTs) were performed. The AUC is shown in **Fig 2B** and the percentage fall from baseline in **Fig 2C**. Insulin sensitivity was significantly impaired in LARNT mice compared to their floxed controls (**p = 0.003** by ANOVA for repeated measures).

### *Arnt* deletion altered hepatic gene expression

*Arnt* mRNA was measured by real time-PCR and was decreased to 20±1% of control levels (p<0.00001, **Fig 2D**), confirming deletion efficiency. Consistent with ARNT being a transcription factor, there were a number of changes in liver gene expression in LARNT mice. Expression of the glucose transporters 1 (*Glut1*) was decreased and expression of the transcription factor Carbohydrate responsive element binding protein (*Chrebp)* was increased (**Fig 2D**). When the rate limiting enzymes in gluconeogenesis were examined, glucose 6-phosphatase *(G6Pase)* was increased by ~60% while a more modest rise was observed in the case of phosphoenolpyruvate carboxykinase (*Pepck)* expression (~20%, p = 0.07, **Fig 2E**), consistent with the finding of increased glucose production after pyruvate. These changes were accompanied by significant decreases in mRNA for insulin receptor (*Insr*) and insulin receptor substrate 2 (*Irs2*), and trends to decreased insulin receptor substrate 1 (*Irs1*) and *Akt2* (**Fig 2E**).

In line with increased *Chrebp* expression LARNT mice had significantly increased levels of lipogenic genes Steroyl Co-A-desaturase 1 (*Scd1*) and HMGCoA-synthase (*Hmgs*) as well as a lesser rise in fatty-acid synthase (*Fas*) (**Fig 2F**). No changes were present in HMGCoA-reductase (*Hmgr*) (**Fig 2F**) or other lipid regulatory genes tested (**Fig 2G**).

### Liver histology in LART mice did not differ from controls

**Fig 3:**
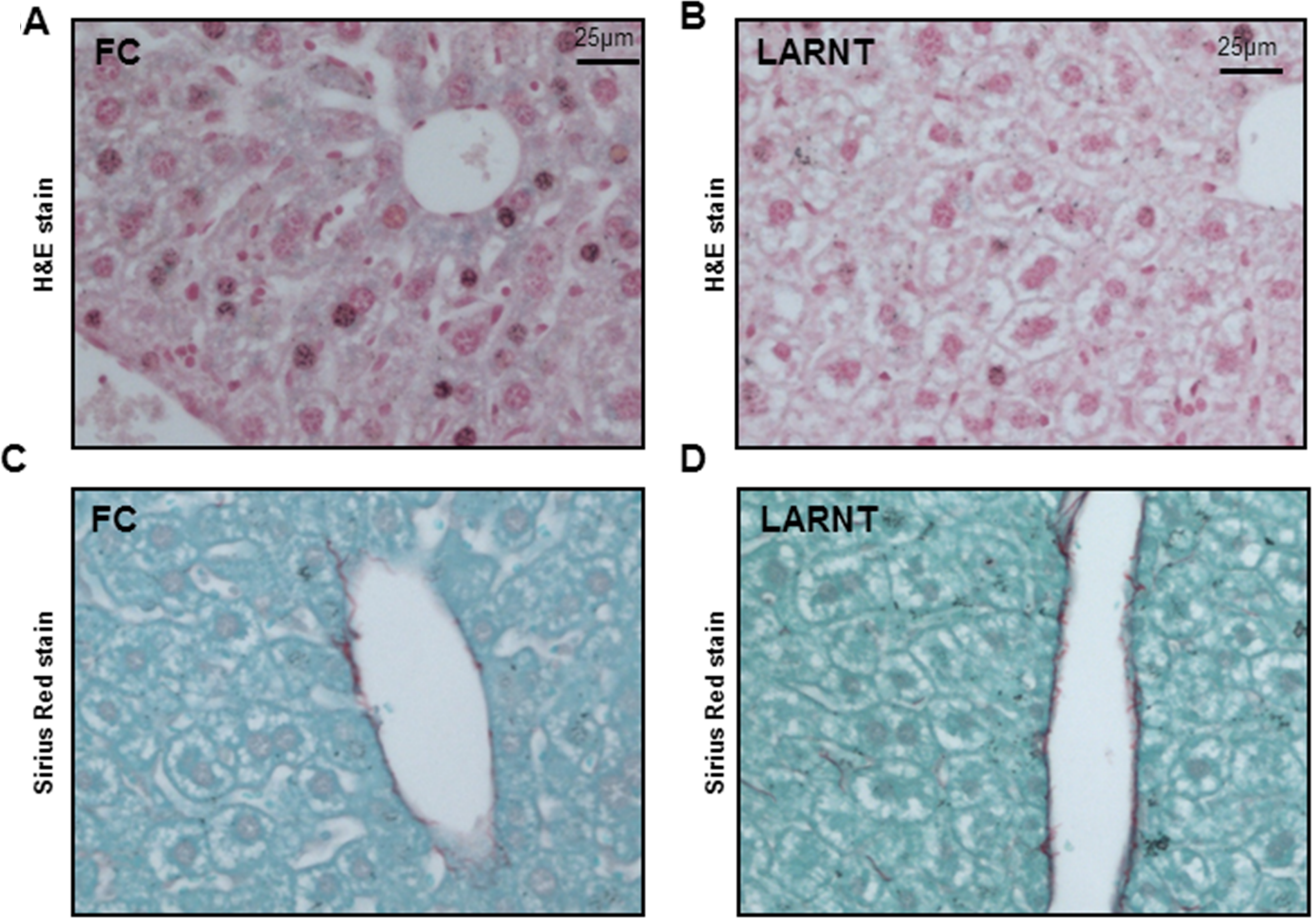
Haematoxylin and Eosin staining of liver sections from fasted floxed control (FC) mice **(A)** and LARNT **(B)** mice. Sirius Red staining from FC **(C)** and LARNT **(D)** mice.

Histological sections from LARNT and control livers were stained with H&E or Sirius red. There were no obvious changes in liver histology as assessed by H&E (**Fig 3A and 3B**), nor Sirius red where red indicates collagen (**Fig 3C and 3D**).

### Hepatic lipid handling and ATP were altered in LARNT mice

**Fig 4:**
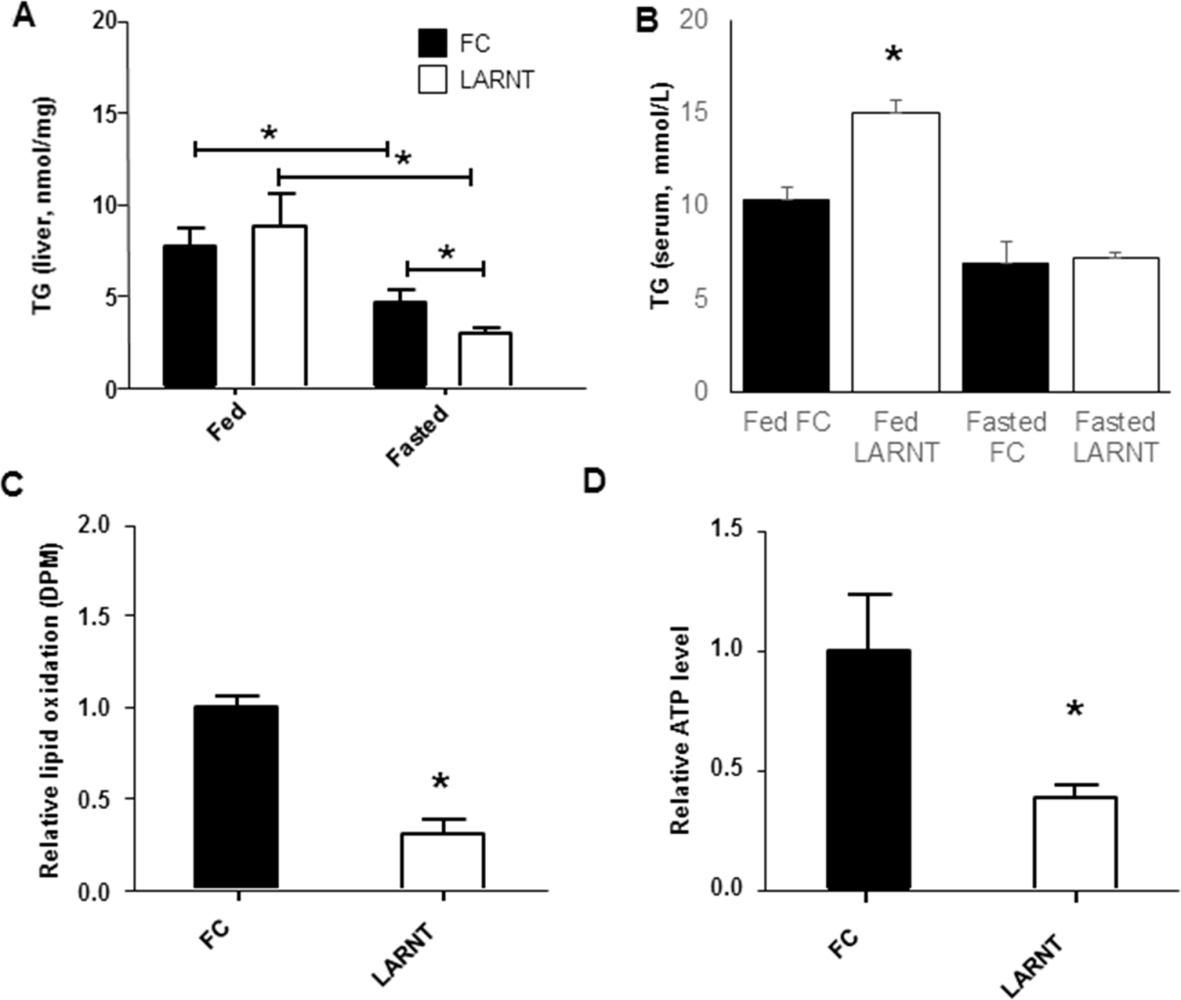
Triglyceride (TG) handling and ATP in LARNT mice. **(A)** Liver TG in FC and LARNT male mice before (Fed) and after a 16hr fast (Fasted). **(B)** TG in serum 6 hours after feeding (Fed) or after a 16 hour fast (Fasted). **(C)** Lipid oxidation in isolated hepatocytes from FC or LARNT animals. (**D)** Whole liver ATP levels in fasted LARNT compared to FC animals. Columns indicate mean±SEM. * = p<0.05. N = 5–6 per group.

There was no significant difference in liver triglyceride content in fed LARNT mice (**Fig 4A**). In both LARNT and FC mice, liver triglyceride was significantly lower in livers of fasted mice than fed mice (**Fig 4A**). However, the decrease between fed and fasting states was greater in LARNT mice, and they had a resulting 26% lower fasting hepatic triglyceride content (**Fig 4A**). We next assessed the effect of ARNT deletion on serum triglyceride as increased levels are a feature of the metabolic syndrome. In fasting mice (**Fig 4B**), there was no difference, however, post-prandial levels were significantly higher in LARNTs (~40% increase, p = 0.0187, **Fig 4B**). To further investigate the cause of the reduced triglyceride content after fasting, we assessed rates of lipid oxidation in LARNT hepatocytes. **Fig 4C** shows that lipid oxidation was *reduced* in isolated LARNT hepatocytes. This experiment measures complete oxidation of lipid to produce CO_2_, so may indicate incomplete oxidation.

Interestingly, ATP concentrations in fasted LARNT livers were markedly reduced, and <40% of FC levels (p<0.02, **Fig 4D**).

### The metabolic phenotypes of LHIF1α and LHIF2α mice were comparable to their floxed control littermates

**Fig 5:**
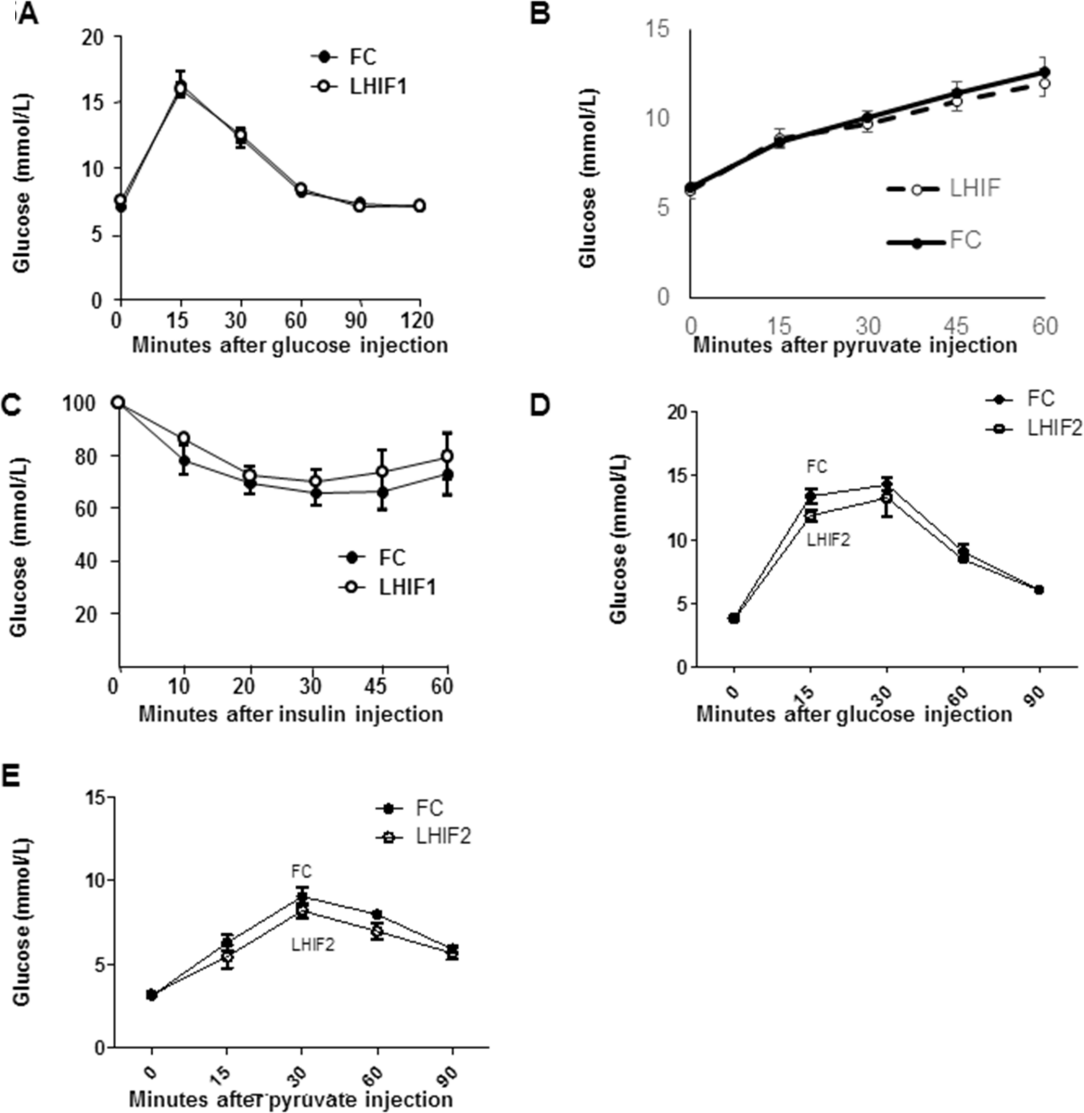
Hepatocyte HIF1-α and hepatocyte HIF2-α deletion have no effects on metabolism in mice on chow diet. Results for floxed controls (FC) are shown in black and LHIF1 or LHIF2 in white circles. (**A**) Glucose tolerance in LHIF mice at 16 weeks. (**B**) Pyruvate tolerance in LHIF mice at 18 weeks. (**C**) Insulin tolerance test in LHIF mice at 19 weeks. (**D**) Glucose tolerance in LHIF2 mice. (**E**) Pyruvate challenge in LHIF2 mice. Mean±SEM is shown, N = 4-5/group.

ARNT functions as a hetero-dimer with another bHLH-PAS transcription factor family member. To test whether HIF-1α or HIF-2α was an important partner for glucose homeostasis in hepatocytes, LHIF1 and LHIF2 mice were created and compared to their respective floxed control littermates (FC). Surprisingly, LHIF1 mice had equivalent fasting glucose levels and glucose tolerance (**Fig 5A**). Their body weight was also not altered, at 25.8±0.9g versus 26.5±1.6g. The kinetics of the response of LHIF1 mice to pyruvate injection was altered (**Fig 5B**), however the area under the curve (AUC) was not different (p = 0.457). LHIF1 mice did not have altered whole-body insulin tolerance (ITT, p>0.5, **Fig 5C**). Likewise LHIF2 mice showed no significant changes in GTT or PCT (**Fig 5D and 5E**).

## Conclusions

Hepatocyte *Arnt* deletion resulted in increased fasting glucose, impaired glucose tolerance, increased glucose production after pyruvate challenge, impaired insulin sensitivity and increased post-prandial serum TG. These changes are similar to those seen in people with T2D and the metabolic syndrome. Hepatocyte HIF-1α and HIF2α deletion had no significant effect on metabolism. Assessment of liver ATP and TG content showed that both were significantly reduced in fasted LARNT mice.

People with T2D have increased HGP [6]. PEPCK and G6Pase are critical enzymes in gluconeogenesis [35, 36] and PEPCK is increased in T2D [37]. G6Pase catalyses the final step in gluconeogenesis and is increased in diabetic animals [36, 38]. The increase in *G6Pase* and trend to increased *Pepck* observed in LARNT mice is consistent with a role for ARNT in the regulation of HGP.

It has recently been demonstrated by magnetic resonance spectroscopy that both ATP and flux through ATP (fATP) are reduced in the livers of T2D patients compared to age matched controls [39, 40]. Further, liver ATP correlated with hepatic insulin sensitivity even after controlling for hepatocyte lipid content [40]. We found that hepatic *Arnt* deletion in LARNT animals resulted in substantially decreased liver ATP and this reduction in ATP was accompanied by an increase in HGP.

We previously reported an acute model of *Arnt* ablation using adenoviral delivery of Cre-recombinase [29]. In common with short-term deletion, long-term loss of *Arnt* also led to alterations in gluconeogenic and lipogenic mRNAs in the liver, increased HGP and deterioration in glucose tolerance. These experiments provide confirmation of these findings using a genetic model.

It was interesting to see that these alterations do not occur in LHIF1α mice, or HIF2α mice. ARNT is a class I member of the bHLH-PAS family, and usually functions in a heterodimer with a class II member. Class II members including HIF1α, HIF2α, AhR, BMAL1 and some of the clock/ circadian rhythm protein. With HIF1α and HIF2α deletion using the same Cre-driver (albumin-Cre) not causing the same effects, another partner must be involved. Based on known effects of AhR, including a ~23% incidence of diabetes in AhR null mice, we speculate that the important ARNT-partner for glucose regulation in hepatocytes may be AhR.

Long term *Arnt* deletion resulted in elevated postprandial serum TG which was not observed after short term deletion. The question of what actually occurs to ARNT’s partners and functionality in diabetic human liver remains unknown. It has however been reported that HIF-1α expression was transiently increased in the mouse livers after high fat/sucrose diet, and expression is also increased in the bile duct ligation model of liver fibrosis and in ethanol induced fatty liver [41–43]. To add to the likelihood that HIF-1α activity is altered in the setting of diabetes evidence suggests that HIF-1α activity is reduced at high glucose concentrations [44–47].

The alterations in lipid handling following *Arnt* deletion are noteworthy in that LARNT mice showed reduced hepatic lipid content on fasting compared to controls but no difference in the fed state. The mechanism for this effect is unclear, but may relate to alterations in lipid handling with fasting. It is consistent with the hepatosteatosis seen with *increasing* ARNT+HIF signaling by hepatic *Vhl* deletion [24, 48–50]. Increasing signaling of the ARNT + HIF-2α pair has been suggested to be important for liver lipid accumulation [49, 50]. The role of ARNT + HIF-1α is less clear with conflicting studies reported [42, 43]. Perhaps paradoxically, given greater falls in liver and serum triglyceride with fasting in LARNT mice, LARNT mice had reduced lipid oxidation. The technique for measurement of lipid oxidation measures complete oxidation, detecting CO2 at the end of the pathway. It is conceivable that in LARNT mice this process is inefficient, resulting in higher lipid use but incomplete utilization. That and impaired glucose uptake / glycolysis gene expression is consistent with the substantially lower ATP content. These changes together would be predicted to increase susceptibility to liver damage with age and stress.

Our data clearly demonstrate for the first time that long-term *Arnt* deletion regulates both hepatic glucose and lipid homeostasis. These animals show increased HGP, reduced hepatic ATP and increased fed serum TG. Increasing ARNT/HIF signaling could potentially improve these parameters in T2D and MetS patients.
